# Analysis of oral microbiota in patients with obstructive sleep apnea-associated hypertension

**DOI:** 10.1038/s41440-019-0260-4

**Published:** 2019-04-11

**Authors:** Chih-Yuan Ko, An-Ke Hu, Dylan Chou, Li-Mei Huang, Huan-Zhang Su, Fu-Rong Yan, Xiao-Bin Zhang, Hua-Ping Zhang, Yi-Ming Zeng

**Affiliations:** 1 Department of Respiratory and Critical Care Medicine, the Second Affiliated Hospital of Fujian Medical University, 362000 Quanzhou, China; 2 Respiratory Medicine Center of Fujian Province, 362000 Quanzhou, China; 3 Key Laboratory of Fujian Medical University, Fujian Province University, 362000 Quanzhou, China; 4 Department of Endocrinology and Metabolism, the Second Affiliated Hospital of Fujian Medical University, 362000 Quanzhou, China; 5 Zhuhai Campus of Zunyi Medical University, Zhuhai, 519090 Guangdong, China; 6 Center for Molecular Diagnosis and Therapy, the Second Affiliated Hospital of Fujian Medical University, 362000 Quanzhou, China

**Keywords:** homocysteine, hypertension, oral microbiota, obstructive sleep apnea-hypopnea syndrome, periodontopathic bacteria

## Abstract

Obstructive sleep apnea–hypopnea syndrome (OSAHS) is an independent risk factor for hypertension (HTN). The oral microbiota plays a pathophysiological role in cardiovascular diseases; however, there are few reports directly investigating and identifying the organisms involved in OSAHS-related HTN. Therefore, this study aimed to identify those organisms. We obtained 139 oral samples and determined the microbiome composition using pyrosequencing and bioinformatic analyses of the 16S rRNA. We examined the fasting levels of cytokines and homocysteine in all participants and analyzed the correlations between the oral microbiota and homocysteine levels. We determined the molecular mechanism underlying HTN by investigating the genetic composition of the strains in the blood. We detected higher relative abundances of *Porphyromonas* and *Aggregatibacter* and elevated proinflammatory cytokines in patients with OSAHS of varying severity compared with individuals without OSAHS; however, the two organisms were not measured in the blood samples from all participants. High levels of specific *Porphyromonas* bacteria were detected in patients with OSAHS with and without HTN, whereas the relative abundance of *Aggregatibacter* was negatively correlated with the homocysteine level. The receiver operating characteristic curve analysis of controls and patients with OSAHS resulted in area under the curve values of 0.759 and 0.641 for patients with OSAHS with or without HTN, respectively. We found that the predictive function of oral microbiota was different in patients with OSAHS with and without HTN. However, there was no direct invasion by the two organisms causing endothelial cell injury, leading to speculation regarding the other mechanisms that may lead to HTN. Elucidating the differences in the oral microbiome will help us understand the pathogenesis of OSAHS-related HTN.

## Introduction

Recent evidence has shown an association between obstructive sleep apnea–hypopnea syndrome (OSAHS), a common sleep breathing disorder, and increased cerebral/cardiovascular morbidity and mortality [1]. Hypertension (HTN) is a systemic disorder and the most prevalent cardiovascular disease (CVD) [2]. Its prevalence among primary and antihypertensive agent-resistant patients with OSAHS is ~65–80% [3, 4], and because blood pressure values also have a significantly positive relationship with the apnea–hypopnea index (AHI), the effects of OSAHS will continue to grow with increasing severity of OSAHS [5]. Hence, more reliable biomarkers and noninvasive screening techniques are needed.

Numerous surveys have implicated microbiota in the pathogenesis of HTN [6–10]. A delicate balance in the microbiota composition is fundamental for maintaining immunity and whole-body homeostasis [11]. Ideally, symbiotic bacteria maintain a constant relationship with the host, which can be compromised by changes in the microbiota composition, termed dysbiosis [12], thereby mediating immune dysregulation [13].

Interestingly, the characteristics of oral bacteria can reflect systemic disorders, including CVDs, coronary heart disease, and HTN [6, 8, 10]. HTN correlates positively with periodontopathic bacterial infections, particularly those caused by *Aggregatibacter* spp. and *Porphyromonas* spp [6, 14]. Furthermore, compared with healthy individuals, patients with OSAHS have considerably higher oral bacterial counts and an increased incidence of periodontitis, including those involving the aforementioned organisms [15]. OSAHS is also characterized by increasing levels of proinflammatory cytokines in the saliva and serum [15], which mediate endothelial cell injury and promote the occurrence and development of HTN [16].

However, the precise effect of oral microbiota on HTN in OSAHS has not yet been clarified. Currently, the entry of periodontal microorganisms and their metabolic products into systemic circulation is regarded as one mechanism that initiates HTN [17, 18]. Therefore, oral bacterial flora is an important factor warranting further study with respect to its role in the development of HTN in OSAHS.

The present study aimed to determine the possible underlying mechanism by evaluating the relationship between oral microbiota and HTN in patients with OSAHS and examining whether the serum of patients with OSAHS contains DNA from *Aggregatibacter* spp. and *Porphyromonas* spp. Furthermore, homocysteine (HCY) levels are associated with the risk of CVD and HTN in OSAHS; [19, 20] therefore, we also assessed the correlation between the oral microbiota and HCY levels.

## Materials and methods

### Subjects

In total, 139 subjects were recruited and underwent a full night (from 2200 to 0800 hours) of polysomnography (PSG; SOMNOscreen™ plus (PSG^+^), SOMNOmedics GmbH, Randersacker, Germany) conducted by technologists in a sleep laboratory at the Department of Respiratory and Critical Care Medicine. Fasting blood and oral mucosal (involving buccal mucosa, tongue, soft palate, and hard palate) samples were collected the following morning. The present study was approved by the Second Affiliated Hospital of Fujian Medical University’s Institutional Review Board with the certificate number IRB No. 2017–78.

### OSAHS assessments

PSG was performed with a computerized polysomnographic system, which simultaneously recorded electrocardiography, electroencephalography, electromyography, electrooculography, and dynamic blood pressure measurements, including systolic blood pressure (SBP) and diastolic blood pressure (DBP). A high blood pressure was defined as SBP ≥ 140 mmHg and/or DBP ≥ 90 mmHg or self-reported use of medications for HTN [4]. Following the examination, AHI was calculated as the total number of episodes of apnea (continuous cessation of airflow for at least 10 s) and hypopnea (reduction in airflow for ≥10s with oxygen desaturation of ≥4%) by dividing the total sleep period by the number of episodes, according to the diagnostic criteria of the American Academy of Sleep Medicine. Adults with AHI ≤5 were used as controls; thus, 5< AHI ≤15 was considered to indicate mild OSAHS and AHI ≥15 was considered to indicate moderate-to-severe OSAHS [5].

### Examination of HCY

The fasting HCY levels were examined with the Automatic Biochemical Analyzer (TBA-120 FR, Toshiba, Japan) and HCY assay kit (Yong He Sun Biotech. Ltd., Hunan, China), using the enzymatic cycling method.

### Cytokine analysis

Interleukin (IL)-2, IL-4, IL-6, IL-10, tumor necrosis factor alpha, and interferon-gamma (IFN-γ) were assayed with the BD™ Cytometric Bead Array Kit Human Enhanced Sensitivity Flex Set (BD Biosciences, New Jersey, USA), as described previously [21]. The standard coefficient of determination was >0.995.

### Sampling, DNA extraction, and 16S rRNA gene amplification sequencing

Once collected, all fresh oral mucosal samples were stored in a Microbiome Test Kit (G-BIO Biotech, Inc., Hangzhou, China). Magnetic bead isolation was used to extract the genomic DNA with a TIANamp Stool DNA Kit (TIANGEN Biotech (Beijing) Co., Ltd., China), according to the instructions. The NanoDrop™ ND-1000 Spectrophotometer (Thermo Fisher Scientific, Massachusetts, USA) was used to determine the concentration of the extracted DNA, and 1.0% agarose gel electrophoresis with 0.5 mg/mL ethidium bromide was used to confirm the quality.

Isolated fecal DNA was used as a template for polymerase chain reaction amplification (forward primer, 5′-ACTCCTACGGGAGGCAGCAG-3′; reverse primer, 5′-GGACTACHVGGGTWTCTAAT-3′) of the V3 and V4 hypervariable regions of the bacterial 16S ribosomal RNA gene. The 16S target-specific sequence contained adaptor sequences, allowing uniform amplification of a highly complex library for downstream next-generation sequencing on the Illumina MiSeq system (Illumina^®^, Inc., California, USA). Negative DNA extraction controls (lysis buffer and kit reagents only) were amplified and sequenced as contamination controls. The amplicons were normalized, pooled, and sequenced on the Illumina^®^ MiSeq platform using a V3 reagent kit with 2 × 300 cycles per sample and the prepared, imported routine data (samsheet) run in the MiSeq sequence program. After sequencing, the Q30 scores were ≥70%, the cluster passing filter (cluster PF) was ≥80%, and there were at least 30,000 clean tags. Finally, image analysis and base calling were performed using the MiSeq Control Software.

### Porphyromonas gingivalis and Aggregatibacter actinomycetemcomitans measurement in the blood

A magnetic bead isolation kit (Magen, Guangzhou, China) was used to extract genomic DNA from all blood samples, according to the instructions. Using real-time PCR, these samples were then examined for DNA from two different bacterial species: *P. gingivalis* (forward primer, 5’-TGTAGATGACTGATGGTGAAAACC-3’; reverse primer 3′-ACGTCATCCACACCTTCCTC-5′) and *A. actinomycetemcomitans* (forward primer 5′-CTTACCTACTCTTGACATCCGAA-3′; reverse primer 3′-ATGCAGCACCTGTCTCAAAGC-5′). The real-time PCR protocol comprised an initial step of predenaturation at 95 °C for 10 min, followed by 40 denaturation cycles at 95°C for 15 s and annealing at 61.5 °C for 30 s.

### Bioinformatic, predictive function, and statistical analyses

The total sequence data were used to analyze the taxa of the oral mucosal microbiota based on the Quantitative Insights into Microbial Ecology bioinformatic pipeline for performing taxonomy assignments using the operational taxonomic unit method. The Phylogenetic Investigation of Communities by Reconstruction of Unobserved States bioinformatics software package and Kyoto Encyclopedia of Genes and Genomes were used to predict the bacterial metabolic functions.

Differences in oral microbiota were analyzed by the Kruskal–Wallis test, as appropriate. Principal coordinate analysis, based on the Bray–Curtis dissimilarity and R statistics, was also performed. Further data analysis by *t*-test or one-way analysis of variance (ANOVA) was undertaken with SPSS version 19.0 (SPSS Inc., Chicago, IL, USA), and the data are expressed as the mean ± standard deviation (SD). Significant differences within groups were analyzed with ANOVA, followed by post hoc Fisher’s least significant difference corrections for multiple comparisons of normally distributed variance data. We considered a two-sided *p* value <0.05 to be statistically significant. The correlation coefficients between oral microbiota and HCY level were evaluated by Spearman’s correlation.

## Results

### Participants’ characteristics

We enrolled 126 patients with OSAHS (mild (*n* = 35) and moderate-to-severe (*n* = 91)) and 13 controls (Table 1). The age of those with moderate-to-severe OSAHS was significantly higher than that of the controls, and body weight was the highest in the moderate-to-severe OSAHS group. Conversely, the body mass index (BMI) and waist circumferences of the controls were the lowest (Table 1).

**Table 1:** Participant characteristics

	Control	Group1	Group2	F value	*P* value	post-hoc test		
	(*n*=13)	(*n*=35)	(*n*=91)			*P* value		
						C vs G1	C vs G2	G1 vs G2
Gender (male/female)	9/4	31/4	81/10	NA^a^	NA	NA	NA	NA
Age (years, mean ± SD)	35.92 ± 7.69	43.57 ± 12.13	46.66 ± 13.35	4.352	0.015	0.065	0.005	0.221
Height (cm)	167.92 ± 9.07	167.44 ± 7.48	167.42 ± 7.27	0.026	0.975	NA	NA	NA
Weight (kg)	68.40 ± 9.28	72.87 ± 11.29	78.67 ± 13.53	5.398	0.006	0.279	0.007	0.023
Body mass index (kg m^-2^)	24.10 ± 2.33	26.75 ± 4.79	27.68 ± 3.61	5.118	0.007	0.036	0.002	0.227
Waist circumference (cm)	83.85 ± 6.26	93.51 ± 11.27	96.19 ± 8.33	10.884	< 0.001	0.001	< 0.001	0.137
Hip circumference (cm)	97.81 ± 4.10	99.00 ± 7.77	100.54 ± 6.15	1.471	0.233	NA	NA	NA
Homocysteine (umol/L)	11.29 ± 3.13	15.43 ± 6.67	16.27 ± 8.07	2.572	0.080	NA	NA	NA
Sleep efficiency (%)	68.80 ± 17.65	68.74 ± 17.92	72.08 ± 15.17	0.668	0.515	NA	NA	NA
Arousal index (events/h)	3.57 ± 1.87	3.35 ± 1.71	3.23 ± 2.44	0.148	0.863	NA	NA	NA
Apnea-hypopnea index (events/h)	1.83 ± 1.34	9.19 ± 3.13	46.44 ± 21.66	78.109	< 0.001	0.202	< 0.001	< 0.001
Hypopnea index (events/h)	1.21 ± 0.91	6.27 ± 3.19	17.16 ± 11.19	29.050	< 0.001	0.094	< 0.001	< 0.001
Mean SpO_2_ (%)	95.62 ± 1.12	94.86 ± 1.46	92.81 ± 2.42	18.299	< 0.001	0.273	< 0.001	< 0.001
Lowest SpO_2_ (%)	90.62 ± 3.28	85.11 ± 4.71	75.30 ± 8.79	37.327	< 0.001	0.027	< 0.001	< 0.001
Average systolic blood pressure (mmHg)	109.15 ± 19.94	112.94 ± 32.43	128.76 ± 25.36	6.253	0.003	0.665	0.015	0.004
Average diastolic blood pressure (mmHg)	74.31 ± 11.43	77.83 ± 22.05	86.31 ± 14.80	13.28	< 0.001	0.116	0.001	0.024

^a^N/A: not analyzed. Control: apnea–hypopnea index (AHI) ≤ 5 (non-OSAHS), Group 1: 5 < AHI ≤ 15 (mild OSAHS with/without hypertension), Group 2: AHI > 15 (moderate-to-severe OSAHS with/without hypertension); the statistical analysis was performed with Fisher’s least significant difference test

The AHI, hypopnea index, and mean SpO_2_ were the highest in patients with moderate-to-severe OSAHS, whereas the lowest SpO_2_ was in those with mild OSAHS (Table 1). Both the average SBP and DBP were the highest in patients with moderate-to-severe OSAHS (Table 1). We also examined patients with OSAHS and HTN, dividing them into five groups (see supplementary Table 1 for the detailed data).

### Changes in taxa among groups

Simpson’s diversity index detected no statistically significant differences in community richness and diversity among the control group and other groups (see supplementary Figure 1 for details).

**Fig. 1:**
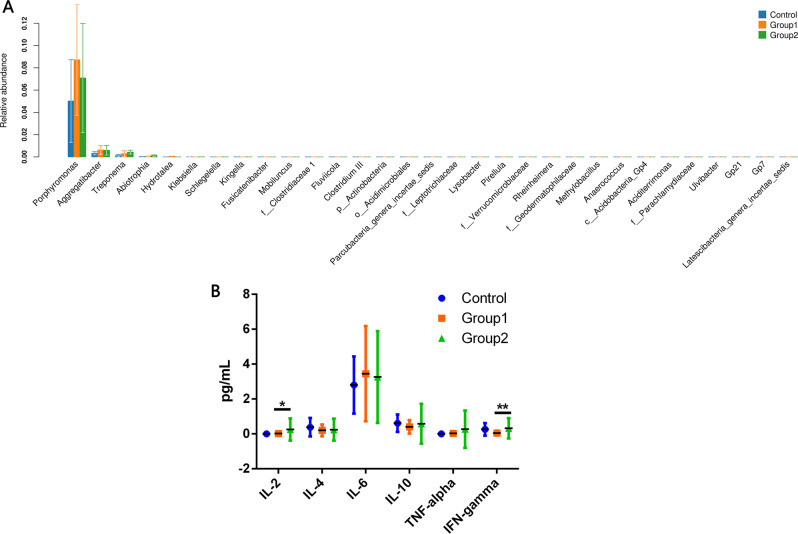
Differences in the fecal microbiomes and cytokine levels in patients with obstructive sleep apnea–hypopnea syndrome (OSAHS) and the control group. There were significant differences in the fecal microbiome at the genera level (**a**), and the statistical analysis was performed with the Kruskal–Wallis test. Proinflammatory cytokines in patients with OSAHS (**b**); the statistical analysis was performed with Fisher’s least significant difference test. *IL* interleukin, *TNF* tumor necrosis factor, *IFN* interferon. Control: apnea–hypopnea index (AHI) ≤5 (non-OSAHS), Group 1: 5< AHI ≤15 (mild OSAHS with/without hypertension), Group 2: AHI >15 (moderate-to-severe OSAHS with/without hypertension). *P<0.05, **P<0.01

The relative abundances of the *Porphyromonas* and *Aggregatibacter* genera in Group 1 were significantly higher than those in the controls. *Treponema*, *Abiotrophia*, *Hydrotalea*, and *Schlegelella* were also significantly more abundant in Group 2 than in the controls. However, *Kingella*, *Fusicatenibacter*, *Mobiluncus*, *f__Clostridiaceae 1*, *Fluviicola*, *Clostridium III*, *p__Actinobacteria*, *o__Acidimicrobiales*, *Parcubacteria_genera_incertae_sedis*, *f__Leptotrichiaceae*, *Lysobacter*, *Pirellula*, *f__Verrucomicrobiaceae*, *Rheinheimera*, *f__Geodermatophilaceae*, *Methylobacillus*, *Anaerococcus*, *c__Acidobacteria_Gp4*, *Aciditerrimonas*, *f__Parachlamydiaceae*, *Ulvibacter*, *Gp21*, *Gp7*, and *Latescibacteria_genera_incertae_sedis* genera were more abundant in the controls than in those with OSAHS (Fig. 1a; see supplementary Table 2 for detailed statistics).

**Fig. 2:**
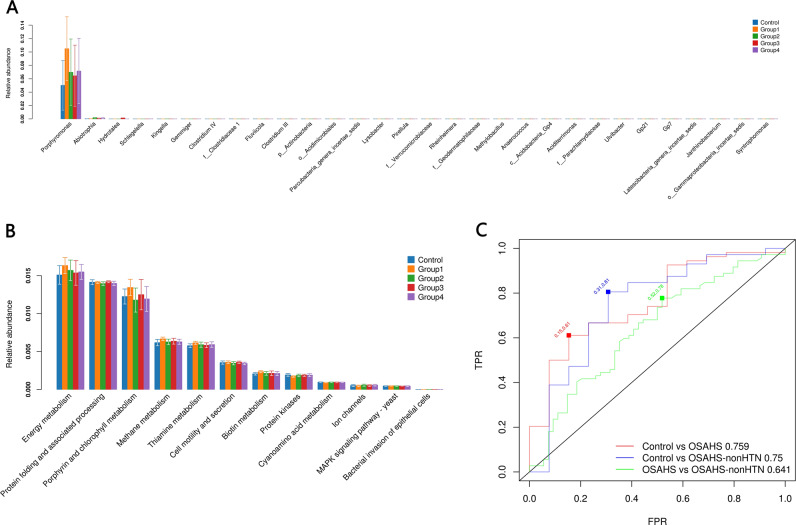
Taxonomic differences in the fecal microbiota in the control group and patients with obstructive sleep apnea–hypopnea syndrome (OSAHS) with and without hypertension. There were significant differences in the fecal microbiome at the genera level (**a**). Kyoto Encyclopedia of Genes and Genomes pathways are shown for the fecal microbiome (**b**). Patients with OSAHS could be separated from controls with an area under the receiver operating characteristic curve (ROC–AUC) of 0.759; patients with OSAHS without HTN could be distinguished from the controls with an ROC–AUC of 0.750; and patients with OSAHS could be distinguished from patients with OSAHS without HTN with an ROC–AUC of 0.641 (**c**). The statistical analysis was performed with the Kruskal–Wallis test. Control: apnea–hypopnea index (AHI) ≤5 (non-OSAHS), Group 1: 5< AHI ≤15 (mild OSAHS without hypertension), Group 2: AHI >15 (moderate-to-severe OSAHS without hypertension), Group 3: mild OSAHS with hypertension, Group 4: moderate-to-severe OSAHS with hypertension. FPR: false positive rate; TPR: true positive rate

The relative abundance of the *Porphyromonas* genera was significantly higher in Group 1 than in the controls, as were the relative abundances of *Abiotrophia* in Group 2 and Group 4 and *Hydrotalea* and *Kingella* in Group 4. *Gemmiger* was significantly more abundant in Group 2 than in Group 1. *Gammaproteobacteria_incertae_sedis* and *Syntrophomonas* were significantly more abundant in Group 3 than in the controls. However, *Schlegelella*, *f__Clostridiaceae 1*, *Fluviicola, Clostridium III*, *p__Actinobacteria*, *o__Acidimicrobiales*, *Parcubacteria_genera_incertae_sedis*, *Lysobacter*, *Pirellula*, *f__Verrucomicrobiaceae*, *Rheinheimera*, *f__Geodermatophilaceae*, *Methylobacillus*, *Anaerococcus*, *c__Acidobacteria_Gp4*, *Aciditerrimonas*, *f__Parachlamydiaceae*, *Ulvibacter*, *Gp21*, *Gp7*, *Latescibacteria_genera_incertae_sedis*, and *Janthinobacterium* genera were more abundant in the controls than in those with OSAHS (Fig. 2a; see supplementary Table 3 for detailed statistics).

### Cytokine analysis

The levels of IL-2 [F(2,138) = 3.211, *p* = 0.043] and IFN-γ [F(2,138) = 3.987, *p* = 0.021] were significantly higher among patients with OSAHS than among the controls; post hoc testing indicated higher levels among those with AHI ≥15 than among those with AHI <15 (Fig. 1b).

### Predictive function analysis

The relative abundances of energy and biotin metabolism were significantly higher in Group 1 than in the controls, whereas methane and thiamin metabolism were significantly higher in Group 1 than in the controls and Group 2. Protein folding and associated processing were significantly higher in Group 3 than in Group 2. The relative abundances of cell motility and secretion, protein kinases, cyanoamino acid metabolism, and ion channels were significantly higher in Group 4 than in Group 1. Conversely, mitogen-activated protein kinase signaling pathway-yeast was significantly higher in Group 1 than in Group 4, and porphyrin and chlorophyll metabolism was significantly higher in Group 1 than in both Group 4 and Group 2. Bacterial invasion of epithelial cells was significantly higher in Group 2 than in the controls (Fig. 2b; detailed statistics are presented in supplementary Table 4).

### Oral microbiota discrimination predictive model

To select predictive features of oral microbiomes, we discriminated between patients with OSAHS and those with OSAHS without HTN and controls with areas under the receiver operating characteristic curve (ROC–AUCs) of 0.759 and 0.750, respectively, and discriminated patients with OSAHS from those with OSAHS without HTN with an ROC–AUC of 0.641 (Fig. 2c).

### Quantitative detection of 18S rDNA genes of *P. gingivalis* and *A. actinomycetemcomitans* in serum

Neither *P. gingivalis* nor *A. actinomycetemcomitans* 18S recombinant DNA genes were detected in the 139 samples (see supplementary Figure 2 for details).

### Correlation between oral microbiota and homocysteine levels

**Fig. 3:**
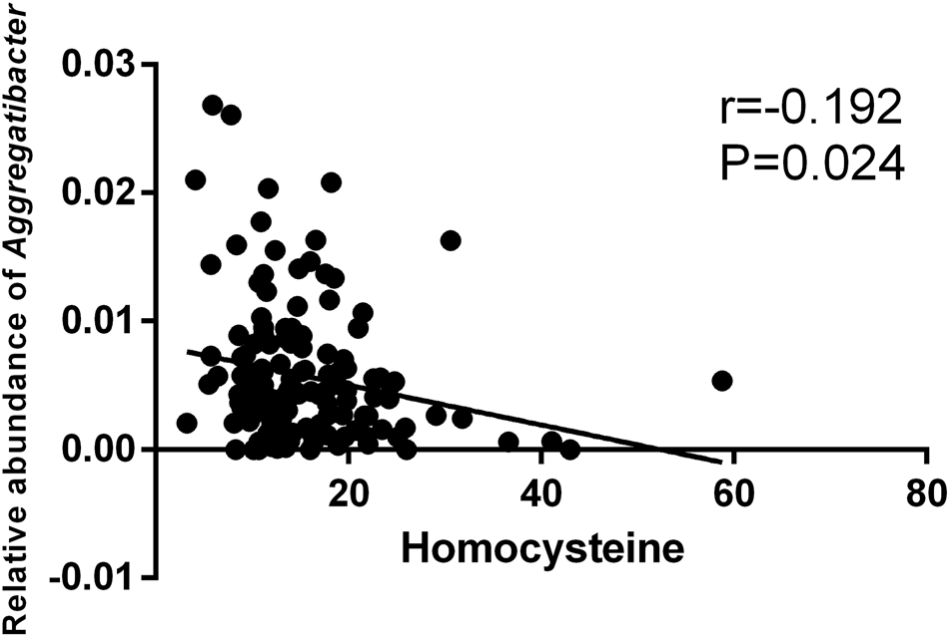
The relative abundance of *Aggregatibacter* was negatively correlated with the homocysteine level

The correlations between HCY levels and the relative abundance of microbiota components are shown in Fig. 3; a correlation existed between HCY levels and the relative abundance of *Aggregatibacter* (r = −0.192, *p* = 0.024) (see supplementary Table 5 for details).

## Discussion

We conducted 16S rRNA pyrosequencing and bioinformatic analyses to compare the bacterial composition of oral samples from patients with OSAHS and HTN of varying severity. This approach enabled a relatively comprehensive description of the oral microbiome, with high levels of the *Porphyromonas* and *Aggregatibacter* genera being detected, accompanied by elevated levels of proinflammatory cytokines. Subsequently, data revealed that specific bacteria in the *Porphyromonas* genera were detected at high levels in patients with OSAHS with and without HTN and that the relative abundance of *Aggregatibacter* negatively correlated with the HCY levels. Moreover, the validated cutoff ROC–AUC values of 0.759 for controls and patients with OSAHS and 0.641 for patients with OSAHS with and without HTN were detected. The functional analyses of the microbiome were also different, confirming that oral microbiota play a pathophysiological role in HTN in those patients.

OSAHS is an independent risk factor for CVDs, such as HTN [17, 22–24]. Most patients with OSAHS and comorbid HTN may develop refractory HTN, requiring a combination treatment with multiple antihypertensive medications, yet still achieving inadequate control of blood pressure [25]. The prevalence of HTN in the general population is ~20%; however, ~50–60% of patients with OSAHS have comorbid HTN, and ~50% of patients with HTN have OSAHS [26]. It has been noted that patients with HTN have elevated oral bacterial counts [27, 28]. Studies have revealed that those who rarely brush their teeth are at a significantly higher risk for HTN than those who brush after each meal; decreasing the frequency of brushing increases the incidence of HTN [27, 28].

The entry of oral microbiota and their metabolic products into systemic circulation is mechanism initiating cardiovascular events [17, 18]. Aoyama et al[6]. compared the bacterial status in saliva and subgingival plaques in patients with periodontitis with and without HTN and found that those with HTN had a significantly higher detection rate of *A. actinomycetemcomitans*. Therefore, periodontopathogenic bacteria (*P. gingivalis*, *Fusobacterium nucleatum*, *Prevotella intermedia*, and *Bacteroides forsythus*) might enter atherosclerotic lesions directly and play a role in plaque formation [29].

In fact, periodontitis, an infectious disease of the tooth-supporting structures caused by oral pathogens, has been found to be a risk factor for CVD [30]. Recently, it has been shown that patients with OSAHS have markedly higher oral bacterial counts than those of healthy individuals, and they present with a relatively higher incidence of periodontitis [15, 31, 32], which is consistent with our findings. We found that *Aggregatibacter* and *Porphyromonas* particularly had substantial effects.

However, the mechanism by which the oral microbiome contributes to CVD and HTN remains unclear. Direct invasion is an easy and fairly simple pathway. Evidence that identical oral bacterial pathogens are present in the subgingival and atherosclerotic plaques [29, 33] suggest that oral bacteria may directly invade atherosclerotic plaques, heart valves, and other cardiovascular tissues, as well as lesions, directly exerting their effects on CVDs. Based on our functional analyses of the microbiome, the bacterial invasion of the functional epithelial cells was significant in patients with OSAHS and HTN. Thus, we investigated whether the DNA of both *P. gingivalis* and *A. actinomycetemcomitans* existed in the blood to confirm the possible pathophysiological mechanisms involving the two organisms in OSAHS-related HTN.

Nevertheless, we found neither *P. gingivalis* nor *A. actinomycetemcomitans* genes in any of the patients. Pathogenic oral bacteria can induce proinflammatory factors [34] that mediate endothelial cell injury, increase vascular reactivity and resistance, inhibit vasodilators, and promote vasoconstrictor synthesis, vascular remodeling, and the occurrence and development of HTN [35]. We suggest that OSAHS-related HTN may result from relevant inflammatory markers in oral bacterial pathogen-induced pathways.

DNA and lipopolysaccharides in oral bacterial pathogens activate nuclear factor-kappa B (NF-κB) and activator protein 1 (AP1), resulting in the increased production of inflammatory factors, which synergize with bacterial cell wall components, activate neutrophils, and produce large amounts of reactive oxygen species (ROS) [16]. Additionally, hypoxia caused by OSAHS causes cell and tissue damage, which may lead to mitochondrial dysfunction, thereby affecting related enzyme systems, such as xanthine oxidase, endothelial nitric oxide (NO) synthase, nicotinamide adenine dinucleotide phosphate, and ROS production. These two pathways, when combined, produce excessive ROS and oxides, such as hydrogen peroxide and hydroxyl radicals, and the peroxides can react with NO to generate more free radicals. This process weakens the vasodilatory function of NO and severely compromises the function of endothelial cells. Furthermore, redox disequilibrium leads to excessive ROS, thereby aggravating the damage. In addition, studies have also shown that ROS participate in transduction in signaling pathways and promote NF-κB or AP1 to produce excessive inflammatory factors, leading to increases in immune cells, monocytes, neutrophils, and lymphocytes, thereby contributing to the generation of more ROS, forming a vicious cycle [36, 37].

On the other hand, it has been noted that HCY levels are associated with an increased risk of metabolic abnormalities and HTN in patients with OSAHS [19]. However, our data revealed a negative correlation between *Aggregatibacter* and HCY levels. Theoretically, a positive correlation exists because *A. actinomycetemcomitans* contains the gene required for HCY synthesis and can thus stimulate HCY production; [38] this is also true for *Porphyromonas* [39]. However, the results of our functional analyses of the microbiome did not show the depletion of vitamin B6, B12, folates, or methionine in relation to HCY metabolism. Thus, future studies should reexamine these questions in subgroups of hypertensive and normotensive patients with OSAHS to assess the endothelial injury attributed to oral microbiome-produced HCY levels. Moreover, a comprehensive analysis of the possible mechanisms underlying oral microbiota-induced OSAHS-related HTN, including immune, inflammatory, and oxidative stress responses, should be performed.

## Conclusions

Overall, we found evidence of changes in the oral microbiota in patients with OSAHS and an association between oral bacteria of the *Porphyromonas* and *Aggregatibacter* genera and OSAHS-linked HTN. Our findings regarding the close association of some oral microbiota with HTN and the clinical characteristics of CVD may also enhance our understanding of the pathogenesis of OSAHS-linked HTN by elucidating the basis for the differences in the oral microbiome. Moreover, this could support effective intervention for, management of, and prognostic evaluations of modifying oral microbiota in patients with OSAHS.

## Supplementary information

Supplementary Table 1

Supplementary Table 2

Supplementary Table 3

Supplementary Table 4

Supplementary Table 5

Supplementary Figure 1

Supplementary Figure 2
