# Supplementary material for: Analysis of oral microbiota in patients with obstructive sleep apnea-associated hypertension
Source: Hypertens Res. 2019 Apr 11;42(11):1692–700. doi: 10.1038/s41440-019-0260-4 (PMC8075895; doi:10.1038/s41440-019-0260-4)
Supplement: Supplementary file 1 — Supplementary Table 1 [file 41440_2019_260_MOESM1_ESM.docx]

**Participant characteristics of OSAHS patients with and without hypertension**

The ages of those with moderate-to-severe OSAHS with hypertension (HTN) patients (Group4) was significantly higher than others. Body weight of Group2 was higher than Control and Group1; this phenomenon is also shown in Group4. Conversely, waist circumference of controls was the lowest. Hip circumference of Group3 was the highest. AHI and hypopnea index of controls was the lowest. However, the mean SpO_2_ and lowest SpO_2_ of controls was the highest (Supplementary Table 1). Both average systolic and diastolic blood pressure (SBP/DBP) were the highest in Group4; both average SBP and DBP of Group1 and Group2 were lower than Group3 and Group4, respectively (Supplementary Table 1).

**Supplementary** **Table 1. Participant characteristics of patients with obstructive sleep apnea-hypopnea syndrome and hypertension.**

|  |  |  |  |  |  |  |  |  |  |  |  |  |  |  |  |  |  |
| --- | --- | --- | --- | --- | --- | --- | --- | --- | --- | --- | --- | --- | --- | --- | --- | --- | --- |
|  | Control | Group1 | Group2 | Group3 | Group4 | F value | *P* value | post-hoc test | | | | | | | | | |
|  |  |  |  |  |  |  |  | *P* value | | | | | | | | | |
|  | (n=13) | (n=20) | (n=34) | (n=15) | (n=57) |  |  | C vs G1 | C vs G2 | C vs G3 | C vs G4 | G1 vs G2 | G1 vs G3 | G1 vs G4 | G2 vs G3 | G2 vs G4 | G3 vs G4 |
| Gender (male/female) | 9/4 | 18/2 | 32/2 | 13/2 | 49/8 | NA^a^ | NA | NA | NA | NA | NA | NA | NA | NA | NA | NA | NA |
| Age (years, mean ± SD) | 35.92±7.69 | 43.60±13.95 | 36.97±9.45 | 43.53±9.66 | 52.44±11.94 | 13.06 | **<0.001** | 0.055 | 0.773 | 0.074 | **<0.001** | **0.037** | 0.985 | **0.003** | 0.465 | **<0.001** | **0.007** |
| Height (cm) | 167.92±9.07 | 167.10±6.24 | 170.17±7.64 | 167.90±9.10 | 165.78±6.57 | 1.941 | 0.107 | NA | NA | NA | NA | NA | NA | NA | NA | NA | NA |
| Weight (kg) | 68.40±9.28 | 69.50±10.42 | 79.87±17.41 | 77.37±11.13 | 77.95±10.70 | 3.692 | **0.007** | 0.807 | **0.006** | 0.062 | **0.015** | **0.004** | 0.069 | **0.011** | 0.523 | 0.483 | 0.874 |
| Body mass index (kg m^-2^) | 24.1±2.33 | 25.09±4.34 | 26.56±3.43 | 28.96±4.58 | 28.35±3.57 | 6.422 | **<0.001** | 0.452 | **0.043** | **<0.001** | **<0.001** | 0.159 | **0.003** | **<0.001** | **0.037** | **0.027** | 0.569 |
| Waist circumference (cm) | 83.85±6.26 | 89.03±10.01 | 93.69±8.20 | 99.5±10.26 | 97.68±8.11 | 10.459 | **<0.001** | 0.091 | **<0.001** | **<0.001** | **<0.001** | 0.055 | **<0.001** | **<0.001** | **0.030** | **0.033** | 0.463 |
| Hip circumference (cm) | 97.81±4.10 | 96.10±7.83 | 99.50±5.70 | 102.87±5.96 | 101.16±6.37 | 3.691 | **0.007** | 0.443 | 0.407 | **0.034** | 0.083 | 0.055 | **0.002** | **0.002** | 0.083 | 0.221 | 0.346 |
| Homocysteine (umol/L) | 11.29±3.13 | 14.66±6.95 | 18.1±10.57 | 16.46±6.37 | 15.17±5.95 | 2.272 | 0.065 | NA | NA | NA | NA | NA | NA | NA | NA | NA | NA |
| Sleep efficiency (%) | 68.80±17.65 | 68.05±14.85 | 74.27±14.72 | 69.67±21.89 | 70.78±15.41 | 0.601 | 0.662 | NA | NA | NA | NA | NA | NA | NA | NA | NA | NA |
| Arousal index (events/h) | 3.57±1.87 | 3.83±1.74 | 2.81±2.17 | 2.70±1.47 | 3.49±2.58 | 1.130 | 0.345 | NA | NA | NA | NA | NA | NA | NA | NA | NA | NA |
| Apnea-hypopnea index (events/h) | 1.83±1.34 | 9.29±3.12 | 47.93±20.76 | 9.07±3.24 | 45.56±22.30 | 38.178 | **<0.001** | 0.241 | **<0.001** | 0.285 | **<0.001** | **<0.001** | 0.971 | **<0.001** | **<0.001** | 0.540 | **<0.001** |
| Hypopnea index (events/h) | 1.21±0.91 | 5.74±2.94 | 15.74±10.89 | 6.97±3.47 | 18.01±11.38 | 14.822 | **<0.001** | 0.172 | **<0.001** | 0.103 | **<0.001** | **<0.001** | 0.698 | **<0.001** | **0.003** | 0.260 | **<0.001** |
| Mean SpO_2_ (%) | 95.62±1.12 | 94.75±1.41 | 93.09±2.17 | 95.00±1.56 | 92.65±2.56 | 9.301 | **<0.001** | 0.254 | **<0.001** | 0.444 | **<0.001** | **0.007** | 0.732 | **<0.001** | **0.004** | 0.343 | **<0.001** |
| Lowest SpO_2_ (%) | 90.62±3.28 | 84.05±5.20 | 74.09±8.60 | 86.53±3.66 | 76.02±8.89 | 19.307 | **<0.001** | **0.016** | **<0.001** | 0.157 | **<0.001** | **<0.001** | 0.340 | **<0.001** | **<0.001** | 0.242 | **<0.001** |
| Average systolic blood pressure (mmHg) | 109.15±19.94 | 102.90±38.35 | 117.12±15.72 | 126.33±14.90 | 135.70±27.51 | 8.190 | **<0.001** | 0.490 | 0.338 | 0.076 | **0.001** | **0.049** | **0.008** | **<0.001** | 0.244 | **0.001** | 0.206 |
| Average diastolic blood pressure (mmHg) | 74.31±11.43 | 70.05±25.17 | 80.38±7.79 | 88.20±10.89 | 89.84±16.81 | 36.812 | **<0.001** | 0.807 | 0.188 | **0.005** | **<0.001** | 0.224 | **0.004** | **<0.001** | **0.038** | **<0.001** | 0.491 |
|  |  |  |  |  |  |  |  |  |  |  |  |  |  |  |  |  |  |

^a^N/A: not analyzed. Control: apnoea-hypopnea index (AHI)≤5 (non-OSAHS), Group1: 5<AHI≤15 (mild-OSAHS without hypertension), Group2: AHI>15 (moderate-to-severe OSAHS without hypertension), Group3: mild-OSAHS with hypertension, Group4: moderate-to-severe OSAHS with hypertension.
