# Supplementary material for: Analysis of oral microbiota in patients with obstructive sleep apnea-associated hypertension
Source: Hypertens Res. 2019 Apr 11;42(11):1692–700. doi: 10.1038/s41440-019-0260-4 (PMC8075895; doi:10.1038/s41440-019-0260-4)
Supplement: Supplementary file 2 — Supplementary Table 2 [file 41440_2019_260_MOESM2_ESM.docx]

**Gut microbial taxa among OSAHS groups**

Detailed statistics represented in Supplementary Table 2 and described in Figure 1A of the Results section.

**Supplementary Table 2. Relative abundances of the fecal taxa at genera level in patients with obstructive sleep apnea-hypopnea syndrome (OSAHS) and controls.**

|  |  |  |  |  |  |  |  |
| --- | --- | --- | --- | --- | --- | --- | --- |
|  | Relative abundance | | |  | *P* | | |
|  | Control | Group1 | Group2 |  | C vs G1 | C vs G2 | G1 vs G2 |
| *Porphyromonas* | 0.0502000±0.0370424 | 0.0873000±0.0497893 | 0.0709000±0.0488799 |  | 0.0492 | 0.5029 | 0.2384 |
| *Aggregatibacter* | 0.0032297±0.0014376 | 0.0062696±0.0039602 | 0.0059861±0.0042971 |  | 0.0245 | 0.0173 | 1.0000 |
| *Treponema* | 0.0011629±0.0010245 | 0.0029707±0.0025108 | 0.0042837±0.0017246 |  | 0.0705 | 0.0337 | 1.0000 |
| *Abiotrophia* | 0.0001635±0.0002402 | 0.0005589±0.0002518 | 0.0012483±0.0006138 |  | 0.0685 | 0.0007 | 0.2061 |
| *Hydrotalea* | 0.0000193±0.0000000 | 0.0006488±0.0000000 | 0.0000048±0.0000000 |  | 0.0681 | 0.0137 | 1.0000 |
| *Klebsiella* | 0.0000888±0.0000628 | 0.0000092±0.0000000 | 0.0000950±0.0000268 |  | 0.1157 | 1.0000 | 0.0290 |
| *Schlegelella* | 0.0000199±0.0000000 | 0.0000000±0.0000000 | 0.0000601±0.0000000 |  | 0.0042 | 0.0152 | 0.9023 |
| *Kingella* | 0.0000758±0.0000267 | 0.0000143±0.0000000 | 0.0000454±0.0000211 |  | 0.0087 | 0.0309 | 0.9083 |
| *Fusicatenibacter* | 0.0000308±0.0000163 | 0.0000137±0.0000000 | 0.0000042±0.0000000 |  | 0.0809 | 0.0349 | 1.0000 |
| *Mobiluncus* | 0.0000171±0.0000000 | 0.0000000±0.0000000 | 0.0000083±0.0000000 |  | 0.0290 | 0.2565 | 0.2887 |
| *f__Clostridiaceae 1* | 0.0000718±0.0000000 | 0.0000000±0.0000000 | 0.0000000±0.0000000 |  | 0.0147 | 0.0064 | 1.0000 |
| *Fluviicola* | 0.0000553±0.0000000 | 0.0000000±0.0000000 | 0.0000000±0.0000000 |  | 0.0147 | 0.0064 | 1.0000 |
| *Clostridium III* | 0.0000497±0.0000000 | 0.0000000±0.0000000 | 0.0000000±0.0000000 |  | 0.0147 | 0.0064 | 1.0000 |
| *p__Actinobacteria* | 0.0000423±0.0000000 | 0.0000000±0.0000000 | 0.0000000±0.0000000 |  | 0.0147 | 0.0064 | 1.0000 |
| *o__Acidimicrobiales* | 0.0000310±0.0000000 | 0.0000000±0.0000000 | 0.0000000±0.0000000 |  | 0.0147 | 0.0064 | 1.0000 |
| *Parcubacteria_genera_incertae_sedis* | 0.0000168±0.0000000 | 0.0000000±0.0000000 | 0.0000017±0.0000000 |  | 0.0035 | 0.0030 | 1.0000 |
| *f__Leptotrichiaceae* | 0.0000059±0.0000000 | 0.0000048±0.0000000 | 0.0000004±0.0000000 |  | 0.0612 | 0.0117 | 1.0000 |
| *Lysobacter* | 0.0000169±0.0000000 | 0.0000000±0.0000000 | 0.0000000±0.0000000 |  | 0.0147 | 0.0064 | 1.0000 |
| *Pirellula* | 0.0000169±0.0000000 | 0.0000000±0.0000000 | 0.0000000±0.0000000 |  | 0.0147 | 0.0064 | 1.0000 |
| *f__Verrucomicrobiaceae* | 0.0000169±0.0000000 | 0.0000000±0.0000000 | 0.0000000±0.0000000 |  | 0.0147 | 0.0064 | 1.0000 |
| *Rheinheimera* | 0.0000085±0.0000000 | 0.0000010±0.0000000 | 0.0000000±0.0000000 |  | 0.0200 | 0.0010 | 1.0000 |
| *f__Geodermatophilaceae* | 0.0000113±0.0000000 | 0.0000000±0.0000000 | 0.0000000±0.0000000 |  | 0.0147 | 0.0064 | 1.0000 |
| *Methylobacillus* | 0.0000113±0.0000000 | 0.0000000±0.0000000 | 0.0000000±0.0000000 |  | 0.0147 | 0.0064 | 1.0000 |
| *Anaerococcus* | 0.0000087±0.0000000 | 0.0000000±0.0000000 | 0.0000000±0.0000000 |  | 0.0147 | 0.0064 | 1.0000 |
| *c__Acidobacteria_Gp4* | 0.0000085±0.0000000 | 0.0000000±0.0000000 | 0.0000000±0.0000000 |  | 0.0147 | 0.0064 | 1.0000 |
| *Aciditerrimonas* | 0.0000085±0.0000000 | 0.0000000±0.0000000 | 0.0000000±0.0000000 |  | 0.0147 | 0.0064 | 1.0000 |
| *f__Parachlamydiaceae* | 0.0000085±0.0000000 | 0.0000000±0.0000000 | 0.0000000±0.0000000 |  | 0.0147 | 0.0064 | 1.0000 |
| *Ulvibacter* | 0.0000083±0.0000000 | 0.0000000±0.0000000 | 0.0000000±0.0000000 |  | 0.0147 | 0.0064 | 1.0000 |
| *Gp21* | 0.0000056±0.0000000 | 0.0000000±0.0000000 | 0.0000000±0.0000000 |  | 0.0147 | 0.0064 | 1.0000 |
| *Gp7* | 0.0000056±0.0000000 | 0.0000000±0.0000000 | 0.0000000±0.0000000 |  | 0.0147 | 0.0064 | 1.0000 |
| *Latescibacteria_genera_incertae_sedis* | 0.0000056±0.0000000 | 0.0000000±0.0000000 | 0.0000000±0.0000000 |  | 0.0147 | 0.0064 | 1.0000 |
|  |  |  |  |  |  |  |  |

Control: apnoea-hypopnea index (AHI)≤5 (non-OSAHS), Group1: 5<AHI≤15 (mild-OSAHS with/without hypertension), Group2: AHI>15 (moderate-to-severe OSAHS with/without hypertension). Statistical analysis was performed by Kruskal–Wallis test.
