# Supplementary material for: Analysis of oral microbiota in patients with obstructive sleep apnea-associated hypertension
Source: Hypertens Res. 2019 Apr 11;42(11):1692–700. doi: 10.1038/s41440-019-0260-4 (PMC8075895; doi:10.1038/s41440-019-0260-4)
Supplement: Supplementary file 3 — Supplementary Table 3 [file 41440_2019_260_MOESM3_ESM.docx]

**Gut microbial taxa among OSAHS patients with hypertension groups**

Detailed statistics represented in Supplementary Table 3 and described in Figure 2A of the Results section.

**Supplementary Table 3. Relative abundances of the fecal taxa at genera level in controls and patients with obstructive sleep apnea-hypopnea syndrome and hypertension.**

|  |  |  |  |  |  |
| --- | --- | --- | --- | --- | --- |
|  | Relative abundance | | | | |
|  | Control | Group1 | Group2 | Group3 | Group4 |
| *Porphyromonas* | 0.0502072±0.0370424 | 0.1050135±0.0477081 | 0.0697887±0.0495789 | 0.0644417±0.0456980 | 0.0715896±0.0488889 |
| *Abiotrophia* | 0.0001635±0.0002402 | 0.0003544±0.0002502 | 0.0017589±0.0004253 | 0.0008596±0.0004592 | 0.0009438±0.0008894 |
| *Hydrotalea* | 0.0000193±0.0000000 | 0.0000000±0.0000000 | 0.0000129±0.0000000 | 0.0015571±0.0000000 | 0.0000000±0.0000000 |
| *Schlegelella* | 0.0000199±0.0000000 | 0.0000000±0.0000000 | 0.0000022±0.0000000 | 0.0000000±0.0000000 | 0.0000947±0.0000000 |
| *Kingella* | 0.0000758±0.0000267 | 0.0000185±0.0000143 | 0.0000969±0.0000399 | 0.0000097±0.0000000 | 0.0000147±0.0000000 |
| *Gemmiger* | 0.0000057±0.0000000 | 0.0000000±0.0000000 | 0.0000197±0.0000170 | 0.0000101±0.0000000 | 0.0000132±0.0000000 |
| *Clostridium IV* | 0.0000000±0.0000000 | 0.0000282±0.0000000 | 0.0000098±0.0000000 | 0.0000000±0.0000000 | 0.0000033±0.0000000 |
| *f__Clostridiaceae 1* | 0.0000718±0.0000000 | 0.0000000±0.0000000 | 0.0000000±0.0000000 | 0.0000000±0.0000000 | 0.0000000±0.0000000 |
| *Fluviicola* | 0.0000553±0.0000000 | 0.0000000±0.0000000 | 0.0000000±0.0000000 | 0.0000000±0.0000000 | 0.0000000±0.0000000 |
| *Clostridium III* | 0.0000497±0.0000000 | 0.0000000±0.0000000 | 0.0000000±0.0000000 | 0.0000000±0.0000000 | 0.0000000±0.0000000 |
| *p__Actinobacteria* | 0.0000423±0.0000000 | 0.0000000±0.0000000 | 0.0000000±0.0000000 | 0.0000000±0.0000000 | 0.0000000±0.0000000 |
| *o__Acidimicrobiales* | 0.0000310±0.0000000 | 0.0000000±0.0000000 | 0.0000000±0.0000000 | 0.0000000±0.0000000 | 0.0000000±0.0000000 |
| *Parcubacteria_genera_incertae_sedis* | 0.0000168±0.0000000 | 0.0000000±0.0000000 | 0.0000000±0.0000000 | 0.0000000±0.0000000 | 0.0000027±0.0000000 |
| *Lysobacter* | 0.0000169±0.0000000 | 0.0000000±0.0000000 | 0.0000000±0.0000000 | 0.0000000±0.0000000 | 0.0000000±0.0000000 |
| *Pirellula* | 0.0000169±0.0000000 | 0.0000000±0.0000000 | 0.0000000±0.0000000 | 0.0000000±0.0000000 | 0.0000000±0.0000000 |
| *f__Verrucomicrobiaceae* | 0.0000169±0.0000000 | 0.0000000±0.0000000 | 0.0000000±0.0000000 | 0.0000000±0.0000000 | 0.0000000±0.0000000 |
| *Rheinheimera* | 0.0000085±0.0000000 | 0.0000000±0.0000000 | 0.0000000±0.0000000 | 0.0000000±0.0000000 | 0.0000000±0.0000000 |
| *f__Geodermatophilaceae* | 0.0000113±0.0000000 | 0.0000000±0.0000000 | 0.0000000±0.0000000 | 0.0000000±0.0000000 | 0.0000000±0.0000000 |
| *Methylobacillus* | 0.0000113±0.0000000 | 0.0000000±0.0000000 | 0.0000000±0.0000000 | 0.0000000±0.0000000 | 0.0000000±0.0000000 |
| *Anaerococcus* | 0.0000087±0.0000000 | 0.0000000±0.0000000 | 0.0000000±0.0000000 | 0.0000000±0.0000000 | 0.0000000±0.0000000 |
| *c__Acidobacteria_Gp4* | 0.0000085±0.0000000 | 0.0000000±0.0000000 | 0.0000000±0.0000000 | 0.0000000±0.0000000 | 0.0000000±0.0000000 |
| *Aciditerrimonas* | 0.0000085±0.0000000 | 0.0000000±0.0000000 | 0.0000000±0.0000000 | 0.0000000±0.0000000 | 0.0000000±0.0000000 |
| *f__Parachlamydiaceae* | 0.0000085±0.0000000 | 0.0000000±0.0000000 | 0.0000000±0.0000000 | 0.0000000±0.0000000 | 0.0000000±0.0000000 |
| *Ulvibacter* | 0.0000083±0.0000000 | 0.0000000±0.0000000 | 0.0000000±0.0000000 | 0.0000000±0.0000000 | 0.0000000±0.0000000 |
| *Gp21* | 0.0000056±0.0000000 | 0.0000000±0.0000000 | 0.0000000±0.0000000 | 0.0000000±0.0000000 | 0.0000000±0.0000000 |
| *Gp7* | 0.0000056±0.0000000 | 0.0000000±0.0000000 | 0.0000000±0.0000000 | 0.0000000±0.0000000 | 0.0000000±0.0000000 |
| *Latescibacteria_genera_incertae_sedis* | 0.0000056±0.0000000 | 0.0000000±0.0000000 | 0.0000000±0.0000000 | 0.0000000±0.0000000 | 0.0000000±0.0000000 |
| *Janthinobacterium* | 0.0000056±0.0000000 | 0.0000000±0.0000000 | 0.0000000±0.0000000 | 0.0000000±0.0000000 | 0.0000000±0.0000000 |
| *o__Gammaproteobacteria_incertae_sedis* | 0.0000000±0.0000000 | 0.0000000±0.0000000 | 0.0000000±0.0000000 | 0.0000049±0.0000000 | 0.0000000±0.0000000 |
| *Syntrophomonas* | 0.0000000±0.0000000 | 0.0000000±0.0000000 | 0.0000000±0.0000000 | 0.0000048±0.0000000 | 0.0000000±0.0000000 |
|  |  |  |  |  |  |

**Supplementary Table 3 continued**

|  |  |  |  |  |  |  |  |  |  |  |
| --- | --- | --- | --- | --- | --- | --- | --- | --- | --- | --- |
|  | *P* | | | | | | | | | |
|  | C  vs G1 | C  vs  G2 | C  vs G3 | C  vs G4 | G1 vs G2 | G1 vs G3 | G1 vs G4 | G2 vs G3 | G2 vs G4 | G3 vs G4 |
| *Porphyromonas* | 0.020 | 1.000 | 1.000 | 1.000 | 0.105 | 0.177 | 0.095 | 1.000 | 1.000 | 1.000 |
| *Abiotrophia* | 0.824 | 0.014 | 0.145 | 0.003 | 1.000 | 1.000 | 0.522 | 1.000 | 1.000 | 1.000 |
| *Hydrotalea* | 0.112 | 0.258 | 1.000 | 0.033 | 1.000 | 1.000 | 1.000 | 1.000 | 1.000 | 1.000 |
| *Schlegelella* | 0.038 | 0.058 | 0.065 | 0.106 | 1.000 | 1.000 | 1.000 | 1.000 | 1.000 | 1.000 |
| *Kingella* | 0.126 | 1.000 | 0.067 | 0.008 | 0.375 | 1.000 | 1.000 | 0.192 | 0.008 | 1.000 |
| *Gemmiger* | 1.000 | 1.000 | 1.000 | 1.000 | 0.026 | 1.000 | 1.000 | 1.000 | 0.326 | 1.000 |
| *Clostridium IV* | 0.506 | 0.408 | 1.000 | 1.000 | 1.000 | 0.414 | 0.425 | 0.314 | 0.216 | 1.000 |
| *f__Clostridiaceae 1* | 0.109 | 0.054 | 0.167 | 0.032 | 1.000 | 1.000 | 1.000 | 1.000 | 1.000 | 1.000 |
| *Fluviicola* | 0.109 | 0.054 | 0.167 | 0.032 | 1.000 | 1.000 | 1.000 | 1.000 | 1.000 | 1.000 |
| *Clostridium III* | 0.109 | 0.054 | 0.167 | 0.032 | 1.000 | 1.000 | 1.000 | 1.000 | 1.000 | 1.000 |
| *p__Actinobacteria* | 0.109 | 0.054 | 0.167 | 0.032 | 1.000 | 1.000 | 1.000 | 1.000 | 1.000 | 1.000 |
| *o__Acidimicrobiales* | 0.109 | 0.054 | 0.167 | 0.032 | 1.000 | 1.000 | 1.000 | 1.000 | 1.000 | 1.000 |
| *Parcubacteria_genera_incertae_sedis* | 0.033 | 0.013 | 0.057 | 0.026 | 1.000 | 1.000 | 1.000 | 1.000 | 1.000 | 1.000 |
| *Lysobacter* | 0.109 | 0.054 | 0.167 | 0.032 | 1.000 | 1.000 | 1.000 | 1.000 | 1.000 | 1.000 |
| *Pirellula* | 0.109 | 0.054 | 0.167 | 0.032 | 1.000 | 1.000 | 1.000 | 1.000 | 1.000 | 1.000 |
| *f__Verrucomicrobiaceae* | 0.109 | 0.054 | 0.167 | 0.032 | 1.000 | 1.000 | 1.000 | 1.000 | 1.000 | 1.000 |
| *Rheinheimera* | 0.420 | 0.011 | 0.051 | 0.005 | 1.000 | 1.000 | 1.000 | 1.000 | 1.000 | 1.000 |
| *f__Geodermatophilaceae* | 0.109 | 0.054 | 0.167 | 0.032 | 1.000 | 1.000 | 1.000 | 1.000 | 1.000 | 1.000 |
| *Methylobacillus* | 0.109 | 0.054 | 0.167 | 0.032 | 1.000 | 1.000 | 1.000 | 1.000 | 1.000 | 1.000 |
| *Anaerococcus* | 0.109 | 0.054 | 0.167 | 0.032 | 1.000 | 1.000 | 1.000 | 1.000 | 1.000 | 1.000 |
| *c__Acidobacteria_Gp4* | 0.109 | 0.054 | 0.167 | 0.032 | 1.000 | 1.000 | 1.000 | 1.000 | 1.000 | 1.000 |
| *Aciditerrimonas* | 0.109 | 0.054 | 0.167 | 0.032 | 1.000 | 1.000 | 1.000 | 1.000 | 1.000 | 1.000 |
| *f__Parachlamydiaceae* | 0.109 | 0.054 | 0.167 | 0.032 | 1.000 | 1.000 | 1.000 | 1.000 | 1.000 | 1.000 |
| *Ulvibacter* | 0.109 | 0.054 | 0.167 | 0.032 | 1.000 | 1.000 | 1.000 | 1.000 | 1.000 | 1.000 |
| *Gp21* | 0.109 | 0.054 | 0.167 | 0.032 | 1.000 | 1.000 | 1.000 | 1.000 | 1.000 | 1.000 |
| *Gp7* | 0.109 | 0.054 | 0.167 | 0.032 | 1.000 | 1.000 | 1.000 | 1.000 | 1.000 | 1.000 |
| *Latescibacteria_genera_incertae_sedis* | 0.109 | 0.054 | 0.167 | 0.032 | 1.000 | 1.000 | 1.000 | 1.000 | 1.000 | 1.000 |
| *Janthinobacterium* | 0.109 | 0.054 | 0.167 | 0.032 | 1.000 | 1.000 | 1.000 | 1.000 | 1.000 | 1.000 |
| *o__Gammaproteobacteria_incertae_sedis* | 1.000 | 1.000 | 0.032 | 1.000 | 1.000 | 0.011 | 1.000 | 0.003 | 1.000 | 0.001 |
| *Syntrophomonas* | 1.000 | 1.000 | 0.032 | 1.000 | 1.000 | 0.011 | 1.000 | 0.003 | 1.000 | 0.001 |
|  |  |  |  |  |  |  |  |  |  |  |

Control: apnoea-hypopnea index (AHI)≤5 (non-OSAHS), Group1: 5<AHI≤15 (mild-OSAHS without hypertension), Group2: AHI>15 (moderate-to-severe OSAHS without hypertension), Group3: mild-OSAHS with hypertension, Group4: moderate-to-severe OSAHS with hypertension. Statistical analysis was performed by Kruskal–Wallis test.
