# Supplementary material for: Analysis of oral microbiota in patients with obstructive sleep apnea-associated hypertension
Source: Hypertens Res. 2019 Apr 11;42(11):1692–700. doi: 10.1038/s41440-019-0260-4 (PMC8075895; doi:10.1038/s41440-019-0260-4)
Supplement: Supplementary file 4 — Supplementary Table 4 [file 41440_2019_260_MOESM4_ESM.docx]

**Predictive function analysis**

Detailed statistics represented in Supplementary Table 4 and described in Figure 2B of the Results section.

**Supplementary Table 4. Significant Kyoto Encyclopedia of Genes and Genomes pathways were shown for the faecal microbiome.**

|  |  |  |  |  |  |  |  |  |  |  |  |  |  |  |  |  |
| --- | --- | --- | --- | --- | --- | --- | --- | --- | --- | --- | --- | --- | --- | --- | --- | --- |
|  | Relative abundance | | | | |  | *P* | | | | | | | | | |
|  | Control | Group1 | Group2 | Group3 | Group4 |  | C vs G1 | C vs G2 | C vs G3 | C vs G4 | G1 vs G2 | G1 vs G3 | G1 vs G4 | G2 vs G3 | G2 vs G4 | G3 vs G4 |
| Energy metabolism | 0.0150944±0.0012203 | 0.0163362±0.0010504 | 0.0156996±0.0013598 | 0.0153640±0.0016262 | 0.0154980±0.0009791 |  | 0.048 | 1.000 | 1.000 | 1.000 | 0.571 | 0.480 | 0.098 | 1.000 | 1.000 | 1.000 |
| Protein folding and associated processing | 0.0141401±0.0003235 | 0.0141215±0.0001346 | 0.0139494±0.0002464 | 0.0141934±0.0001282 | 0.0139873±0.0002788 |  | 1.000 | 0.236 | 1.000 | 0.663 | 0.441 | 1.000 | 1.000 | 0.005 | 1.000 | 0.017 |
| Porphyrin and chlorophyll metabolism | 0.0122523±0.0009876 | 0.0134442±0.0010790 | 0.0117841±0.0015844 | 0.0124964±0.0019960 | 0.0119349±0.0016363 |  | 0.074 | 1.000 | 1.000 | 1.000 | 0.004 | 0.502 | 0.004 | 1.000 | 1.000 | 1.000 |
| Methane metabolism | 0.0061696±0.0004117 | 0.0066684±0.0002258 | 0.0062631±0.0003627 | 0.0063549±0.0003816 | 0.0062807±0.0003374 |  | 0.002 | 1.000 | 1.000 | 1.000 | 0.000 | 0.104 | 0.000 | 1.000 | 1.000 | 1.000 |
| Thiamine metabolism | 0.0057650±0.0002497 | 0.0061429±0.0001992 | 0.0058989±0.0003418 | 0.0058251±0.0003129 | 0.0059148±0.0003642 |  | 0.022 | 1.000 | 1.000 | 1.000 | 0.046 | 0.131 | 0.069 | 1.000 | 1.000 | 1.000 |
| Cell motility and secretion | 0.0035358±0.0002158 | 0.0035795±0.0001820 | 0.0034481±0.0002150 | 0.0035660±0.0001612 | 0.0034210±0.0001668 |  | 1.000 | 1.000 | 1.000 | 1.000 | 0.202 | 1.000 | 0.018 | 1.000 | 1.000 | 0.619 |
| Biotin metabolism | 0.0020564±0.0001687 | 0.0023313±0.0001387 | 0.0021116±0.0002424 | 0.0021250±0.0003058 | 0.0021084±0.0002637 |  | 0.015 | 1.000 | 1.000 | 1.000 | 0.035 | 0.267 | 0.007 | 1.000 | 1.000 | 1.000 |
| Protein kinases | 0.0019126±0.0001875 | 0.0017042±0.0000930 | 0.0018380±0.0001731 | 0.0018546±0.0001396 | 0.0018782±0.0001943 |  | 0.122 | 1.000 | 1.000 | 1.000 | 0.316 | 1.000 | 0.025 | 1.000 | 1.000 | 1.000 |
| Cyanoamino acid metabolism | 0.0009304±0.0000513 | 0.0008996±0.0000293 | 0.0009263±0.0000446 | 0.0009170±0.0000485 | 0.0009260±0.0000352 |  | 0.202 | 1.000 | 1.000 | 1.000 | 0.115 | 1.000 | 0.103 | 1.000 | 1.000 | 1.000 |
| Ion channels | 0.0005079±0.0001360 | 0.0004563±0.0001051 | 0.0005563±0.0001301 | 0.0004965±0.0001091 | 0.0005541±0.0001039 |  | 1.000 | 1.000 | 1.000 | 1.000 | 0.067 | 1.000 | 0.012 | 1.000 | 1.000 | 0.869 |
| MAPK signaling pathway - yeast | 0.0004398±0.0000385 | 0.0004636±0.0000268 | 0.0004320±0.0000422 | 0.0004515±0.0000116 | 0.0004321±0.0000403 |  | 1.000 | 1.000 | 1.000 | 1.000 | 0.410 | 1.000 | 0.043 | 1.000 | 1.000 | 0.272 |
| Bacterial invasion of epithelial cells | 0.0000017±0.0000013 | 0.0000032±0.0000011 | 0.0000044±0.0000018 | 0.0000027±0.0000012 | 0.0000040±0.0000025 |  | 1.000 | 0.047 | 1.000 | 0.087 | 1.000 | 1.000 | 1.000 | 0.313 | 1.000 | 0.577 |
|  |  |  |  |  |  |  |  |  |  |  |  |  |  |  |  |  |

Control: apnoea-hypopnea index (AHI)≤5 (non-OSAHS), Group1: 5<AHI≤15 (mild-OSAHS without hypertension), Group2: AHI>15 (moderate-to-severe OSAHS without hypertension), Group3: mild-OSAHS with hypertension, Group4: moderate-to-severe OSAHS with hypertension. Statistical analysis was performed by Kruskal–Wallis test.
