# Supplementary material for: Analysis of oral microbiota in patients with obstructive sleep apnea-associated hypertension
Source: Hypertens Res. 2019 Apr 11;42(11):1692–700. doi: 10.1038/s41440-019-0260-4 (PMC8075895; doi:10.1038/s41440-019-0260-4)
Supplement: Supplementary file 5 — Supplementary Table 5 [file 41440_2019_260_MOESM5_ESM.docx]

**Correlations between oral microbiota and homocysteine (HCY)**

There was only a correlation between HCY levels and relative abundance of *Aggregatibacter* (r=-0.192, p=0.024) (Supplementary Table 5).

**Supplementary Table 5. Correlations between HCY levels and relative abundance of microbiota.**

|  | *P* value | Rho value |
| --- | --- | --- |
| *Porphyromonas* | 0.801 | 0.022 |
| *Aggregatibacter* | **0.024** | -0.192 |
| *Treponema* | 0.561 | -0.050 |
| *Abiotrophia* | 0.923 | -0.008 |
| *Hydrotalea* | 0.559 | 0.050 |
| *Klebsiella* | 0.515 | 0.056 |
| *Schlegelella* | 0.774 | 0.025 |
| *Kingella* | 0.708 | -0.032 |
| *Fusicatenibacter* | 0.724 | -0.030 |
| *Mobiluncus* | 0.199 | 0.110 |
| *f__Clostridiaceae 1* | 0.327 | -0.084 |
| *Fluviicola* | 0.327 | -0.084 |
| *Clostridium III* | 0.327 | -0.084 |
| *p__Actinobacteria* | 0.921 | -0.008 |
| *o__Acidimicrobiales* | 0.921 | -0.008 |
| *Parcubacteria_genera_incertae_sedis* | 0.410 | 0.070 |
| *f__Leptotrichiaceae* | 0.070 | -0.154 |
| *Lysobacter* | 0.921 | -0.008 |
| *Pirellula* | 0.921 | -0.008 |
| *f__Verrucomicrobiaceae* | 0.921 | -0.008 |
| *Rheinheimera* | 0.490 | -0.059 |
| *f__Geodermatophilaceae* | 0.921 | -0.008 |
| *Methylobacillus* | 0.921 | -0.008 |
| *Anaerococcus* | 0.594 | -0.046 |
| *c__Acidobacteria_Gp4* | 0.921 | -0.008 |
| *Aciditerrimonas* | 0.921 | -0.008 |
| *f__Parachlamydiaceae* | 0.921 | -0.008 |
| *Ulvibacter* | 0.487 | 0.059 |
| *Gp21* | 0.921 | -0.008 |
| *Gp7* | 0.921 | -0.008 |
| *Latescibacteria_genera_incertae_sedis* | 0.921 | -0.008 |
|  |  |  |
