# Supplementary material for: Analysis of oral microbiota in patients with obstructive sleep apnea-associated hypertension
Source: Hypertens Res. 2019 Apr 11;42(11):1692–700. doi: 10.1038/s41440-019-0260-4 (PMC8075895; doi:10.1038/s41440-019-0260-4)
Supplement: Supplementary file 6 — Supplementary Figure 1 [file 41440_2019_260_MOESM6_ESM.docx]

**Supplementary Information**

We obtained 35396±831 clean reads from 13 faecal samples of the controls, 35082±1991 clean reads from 35 faecal samples of the Group1, 34890±2055 clean reads from 91 faecal samples of the Group2. We observed no statistically significant differences between clean reads among the control group and other groups. Good’s coverage for each sample was >98%, indicating that the OTUs identified in each sample showed the majority of bacterial species identified in all samples.

We found no statistically significant differences in OTUs among the control group and other groups (Supplementary Figure 1A). We examined the mean community diversity indices [Chao (Supplementary Figure 1B), Shannon (Supplementary Figure 1C) and Simpson (Supplementary Figure 1D)] after equalising library sizes to the minimum library size by random subtraction. We detected no statistically significant differences in community richness and diversity and Simpson index among the control group and other groups.

**Supplementary Figure 1. Summary of sequencing data.**

Characteristics of sequencing data in the operational taxonomic units (OTUs) (A), the mean community diversity indices [Chao (B), Shannon (C) and Simpson (D)]. Control: apnoea-hypopnea index (AHI)≤5 (non-OSAHS), Group1: 5<AHI≤15 (mild-OSAHS with/without hypertension), Group2: AHI>15 (moderate-to-severe OSAHS with/without hypertension).
