# Supplementary material for: Analysis of oral microbiota in patients with obstructive sleep apnea-associated hypertension
Source: Hypertens Res. 2019 Apr 11;42(11):1692–700. doi: 10.1038/s41440-019-0260-4 (PMC8075895; doi:10.1038/s41440-019-0260-4)
Supplement: Supplementary file 7 — Supplementary Figure 2 [file 41440_2019_260_MOESM7_ESM.docx]

**Quantitative detection of 18S rDNA genes from *Porphyromonas gingivalis* and *Aggregatibacter actinomycetemcomitans* in serum**

Neither *P. gingivalis* nor *A. actinomycetemcomitans* of genes was detected in 139 samples (Supplementary Figure 2).

**
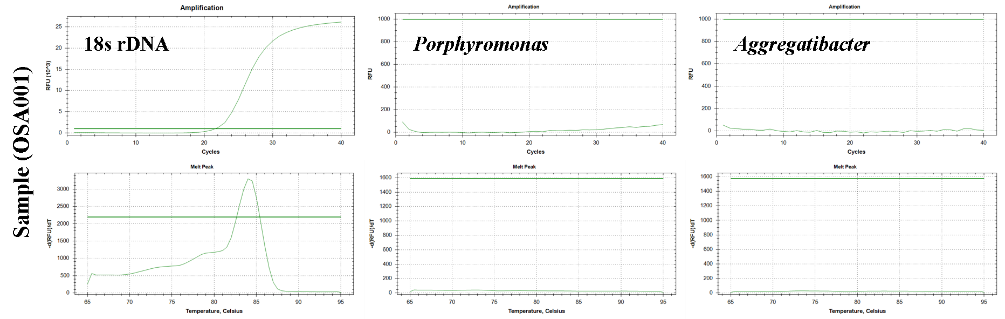
**

**A**

**
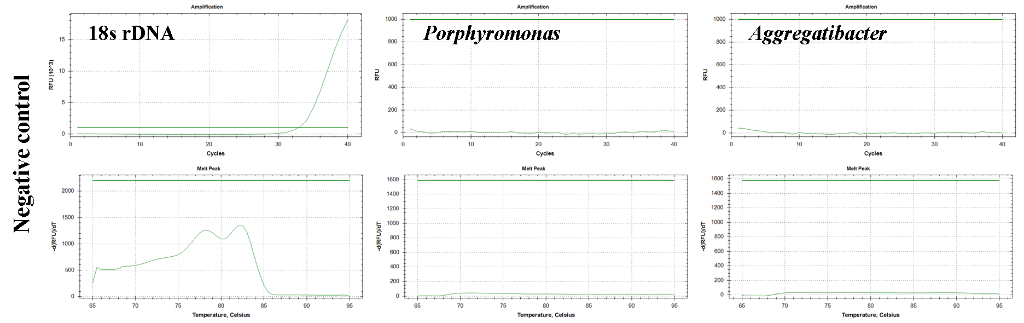

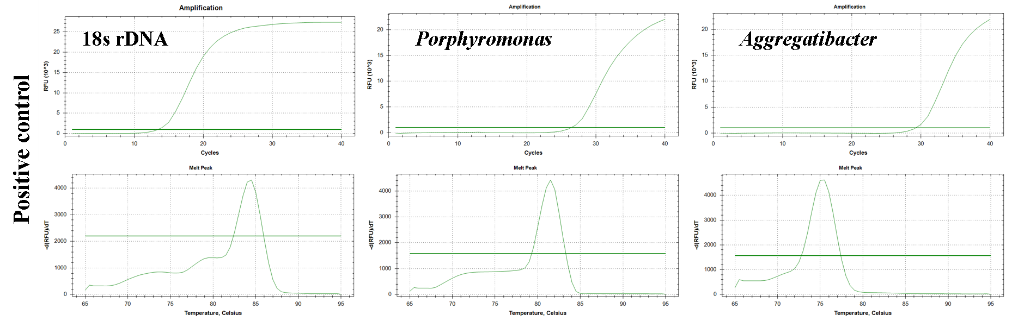
**

**B**

**Supplementary Figure 2. There were not detect *Porphyromonas gingivalis* and *Aggregatibacter actinomycetemcomitans* genes in serum, showing in raw data, taking one sample for example (A),** **quantitative data (B).**

Positive control contains genes of *P. gingivalis* or *A. actinomycetemcomitans* from patient’s oral samples. Negative control contains ddH_2_O. Control: apnea-hypopnea index (AHI)≦5 subjects (n=13). Group1: 5<AHI≦15 mild OSAHS patients with/without hypertension (n=35). Group2: AHI>15 moderate-to-severe OSAHS patients with/without hypertension (n=91).
